# Phase III randomized, placebo‐controlled, double‐blind study of monosialotetrahexosylganglioside for the prevention of oxaliplatin‐induced peripheral neurotoxicity in stage II/III colorectal cancer

**DOI:** 10.1002/cam4.2693

**Published:** 2019-11-13

**Authors:** De‐shen Wang, Zhi‐qiang Wang, Gong Chen, Jie‐wen Peng, Wei Wang, Yan‐hong Deng, Feng‐hua Wang, Jian‐wei Zhang, Han‐lin Liang, Fen Feng, Chuan‐bo Xie, Chao Ren, Ying Jin, Si‐mei Shi, Wen‐hua Fan, Zhen‐hai Lu, Pei‐rong Ding, Feng Wang, Rui‐hua Xu, Yu‐hong Li

**Affiliations:** ^1^ State Key Laboratory of Oncology in South China Collaborative Innovation Center for Cancer Medicine Department of Medical Oncology Sun Yat‐sen University Cancer Center Guangzhou China; ^2^ State Key Laboratory of Oncology in South China Collaborative Innovation Center for Cancer Medicine Department of Colorectal Surgery Sun Yat‐sen University Cancer Center Guangzhou China; ^3^ Zhongshan People's Hospital Zhongshan China; ^4^ The First People's Hospital of Foshan City Foshan China; ^5^ The Sixth Affiliated Hospital of Sun Yat‐sen University Guangzhou China; ^6^ State Key Laboratory of Oncology in South China Collaborative Innovation Center for Cancer Medicine Department of Cancer Prevention Research Sun Yat‐sen University Cancer Center Guangzhou China

**Keywords:** colorectal cancer, EORTCQLQ‐CIPN20, FOLFOX, GM1, neurotoxicity, OIPN, oxaliplatin

## Abstract

**Background:**

Monosialotetrahexosylganglioside (GM1) is a neuroprotective glycosphingolipid that repairs nerves. Oxaliplatin‐based chemotherapy is neurotoxic. This study assessed the efficacy of GM1 for preventing oxaliplatin‐induced peripheral neurotoxicity (OIPN) in colorectal cancer (CRC) patients receiving oxaliplatin‐based chemotherapy.

**Methods:**

In total, 196 patients with stage II/III CRC undergoing adjuvant chemotherapy with mFOLFOX6 were randomly assigned to intravenous GM1 or a placebo. The primary endpoint was the rate of grade 2 or worse cumulative neurotoxicity (NCI‐CTCAE). The secondary endpoints were chronic cumulative neurotoxicity (EORTC QLQ‐CIPN20), time to grade 2 neurotoxicity (NCI‐CTCAE or the oxaliplatin‐specific neuropathy scale), acute neurotoxicity (analog scale), rates of dose reduction or withdrawal due to OIPN, 3‐year disease‐free survival (DFS) and adverse events.

**Results:**

There were no significant differences between the arms in the rate of NCI‐CTCAE grade 2 or worse neurotoxicity (GM1: 33.7% vs placebo: 31.6%; *P = *.76) or neuropathy measured by the EORTC QLQ‐CIPN20 or time to grade 2 neurotoxicity using NCI‐CTCAE and the oxaliplatin‐specific neuropathy scale. GM1 substantially decreased participant‐reported acute neurotoxicity (sensitivity to cold items [*P* < .01], discomfort swallowing cold liquids [*P* < .01], throat discomfort [*P* < .01], muscle cramps [*P* < .01]). The rates of dose reduction or withdrawal were not significantly different between the arms (*P = *.08). The 3‐year DFS rates were 85% and 83% in the GM1 and placebo arms, respectively (*P* = .19). There were no differences in toxicity between the arms.

**Conclusion:**

Patients receiving GM1 were less troubled by the symptoms of acute neuropathy. However, we do not support the use of GM1 to prevent cumulative neurotoxicity. (http://ClinicalTrials.gov number, NCT02251977).

## Graphical abstract

Neuropathy is the most prominent dose‐limiting toxicity of oxaliplatin. Patients receiving Monosialotetrahexosylganglioside (GM1) were less troubled by the symptoms of acute neuropathy. However, we do not support the use of GM1 to prevent cumulative neurotoxicity.

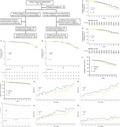

## 1 INTRODUCTION

Several studies have shown that patients with colorectal cancer (CRC) treated with oxaliplatin‐based chemotherapy experience a survival benefit.^1^, ^2^ However, neuropathy is the most prominent dose‐limiting toxicity of oxaliplatin.^3^ Oxaliplatin‐induced peripheral neurotoxicity (OIPN) includes acute neuropathy and chronic neuropathy.^4^ The acute neuropathy commonly consists of transient throat discomfort, sensitivities to touching cold items, discomfort swallowing cold liquids, and muscle cramps, and usually diminish after a few days of exposure. Chronic neuropathy is generally associated with exposure to increasing doses and can cause a peripheral stocking‐glove neuropathy that substantially reduces patients’ quality of life and lasts for months or even years.^5^ During 6 months of treatment with FOLFOX, 43.9%^6^‐47.7%^7^ of patients experienced grade 2 or higher neurotoxicity. Although many efforts have been made to prevent OIPN, the most effective method is to suspend treatment with oxaliplatin.^5^, ^8^ Accordingly, there is an urgent need to identify effective agents that might prevent this common problem for patients receiving oxaliplatin‐based chemotherapy.

The exact mechanisms underlying oxaliplatin‐induced chronic neurotoxicity are undetermined. However, several studies reported that damage to neuronal cell bodies in the dorsal root ganglia (DRG) might be related to the occurrence of oxaliplatin‐induced chronic neurotoxicity.^9^, ^10^, ^11^ The DRG is particularly vulnerable to neurotoxic damage due to the lack of an effective blood‐nerve barrier.^12^ Oxaliplatin may interfere with DNA synthesis in DRG neurons and induce neuronal apoptosis, leading to the predominance of sensory symptoms in OIPN.^9^, ^13^ NGF, a member of the neurotrophin family, is essential for neuronal activity and survival, neurotransmitter expression regulation, synaptic plasticity, neurite outgrowth, and the extension of different types of neurons, including DRG neurons.^10^, ^14^, ^15^ Studies have shown that a decrease in the NGF level is related to the functional injury to the peripheral nervous system caused by platinum‐based drugs.^11^

Monosialotetrahexosylganglioside (GM1) is a glycosphingolipid that is also thought to have neurotrophic factor‐like activity by activating the Trk neurotrophin receptors^16^; it can prevent seizures, Na^+^, and K^+^‐ATPase activity inhibition and oxidative stress induced by glutaric acid^17^ and can enhance the activity of NGF to promote the regeneration and recovery of nerves.^10^ GM1 (Qilu Pharmaceutical Co. Ltd, China) was initially approved for the treatment of vasculogenic or traumatic central nervous impairment and Parkinson disease by the State Food and Drug Administration of China (CFDA). GM1 has also been used in the treatment of diabetic peripheral neuropathy in preclinical animal models, owing to its neurotrophic activities and nerve repair function.^18^ Notably, several prior single‐center retrospective studies reported that GM1 can significantly reduce the incidence of OIPN.^19^, ^20^, ^21^ Therefore, we designed this phase III randomized, placebo‐controlled, double‐blind study to assess the efficacy of GM1 for preventing OIPN in CRC patients who received oxaliplatin‐based adjuvant chemotherapy.

## 2 STUDY DESIGN AND PARTICIPANTS

This trial was a randomized, double‐blind, multi‐centered, placebo‐controlled, phase 3 trial done at 4 academic hospitals in China. Patients considered for recruitment to this trial were over 18 years old with colorectal adenocarcinoma and who, after curative‐intent resection, were scheduled to receive adjuvant chemotherapy with modified FOLFOX6, involving oxaliplatin 85 mg/m^2^ every 2 weeks (12 cycles in total).^6^, ^7^, ^22^ Patients needed to have adequate hematologic parameters and liver and renal function to allow the administration of chemotherapy. Adjuvant chemotherapy was started within 1 month after the resection. A device for central venous access with an implantable port was required to be inserted before starting chemotherapy and protocol treatment.

Patients with a pre‐existing peripheral neuropathy of any grade, who had received prior treatment with neurotoxic chemotherapy such as a taxane, a vinca alkaloid, or cisplatin, who had received any other agent specifically given to treat or prevent neuropathy, or who were considered to be unable to comply with the protocol were not enrolled in the trial. Patients were evaluated every 2 weeks during treatment, every 3 months in the first 3 years after treatment, and then every 6 months up to 5 years after the completion of the study. The protocol was approved by the human research ethics committee at SYSUCC, and all enrolled patients provided appropriate written informed consent (Ethics approval no. B2014‐024‐03).

At the times of their entry into the study, chemotherapy initiation and before each cycle of chemotherapy, a history, physical examination, and laboratory tests (CBC and the levels of creatinine, BUN, AST, ALT, ALP, total bilirubin and serum Na, K, Ca, and Mg) were obtained. At the same time, neurotoxicity assessments were performed by several separate methods.

The primary end point was the rate of grade 2 or worse cumulative neurotoxicity, measured by an investigator using the National Cancer Institute Common Terminology Criteria for Adverse Events (NCI‐CTCAE) version 4.0. The secondary end points were chronic cumulative neurotoxicity measured by the European Organization for Research and Treatment of Cancer Quality of Life Questionnaire‐Chemotherapy‐Induced Peripheral Neuropathy 20 (EORTC QLQ‐CIPN20),^23^ time to grade 2 neurotoxicity according to the NCI‐CTCAE or an oxaliplatin‐specific neuropathy grading scale,^24^ acute neurotoxicity, and the rates of dose reduction or withdrawal due to OIPN in both arms. Acute neurotoxicity was measured with daily questionnaires with a numerical analog scale ranging from 0 to 10 before first dose of mFOLFOX6 and for an additional 6 days after the initiation of each cycle of mFOLFOX6.^5^ At the time of entry into the study and before each cycle of chemotherapy, patients were monitored for AEs, including patient‐reported outcome variables evaluated by questionnaires for laboratory parameters, nausea, vomiting, mucositis, fatigue, hand‐foot syndrome, constipation, and diarrhea.

### 2.1 Randomization and masking

Patients were enrolled by study investigators. A computer program was used to generate the assignment list. Patients were randomly assigned (1:1) to receive intravenous GM1 or an identical‐appearing placebo during chemotherapy. Patients, investigators, and study‐site personnel, those assessing outcomes, and those analyzing the data were masked to their treatment arm.

### 2.2 Procedures

Patients were randomly assigned to receive intravenous GM1 80 mg per day or an identical‐appearing placebo from day 0 to day 4 during each cycle of chemotherapy. For the patients who experienced any clinically significant AE attributed to GM1/the placebo, the GM1/placebo was stopped, and then the patients were observed according to the study protocol. For patients who developed persistent grade 2 sensory neurotoxicity or grade 3 sensory neurotoxicity that resolved within 2 weeks, the dose of oxaliplatin was reduced to 75 mg/m^2^; for patients with persistent grade 3‐4 sensory neurotoxicity, oxaliplatin was discontinued. A 20% dose reduction of fluorouracil and a reduction of oxaliplatin to 75 mg/m^2^ was considered for patients after recovery from grade 3 to 4 nonhematopoietic system toxicity, grade 4 neutropenia or febrile neutropenia or grade 3‐4 thrombocytopenia, and the next dose was delayed until the platelet concentration reached ≥75 × 10^9^/L and the neutrophil concentration reached ≥1.5 × 10^9^/L.

### 2.3 Statistical methodology

Results from the MOSAIC^6^ trial indicated that approximately 40% of patients experience grade 2 or worse chronic neurotoxicity after oxaliplatin‐based chemotherapy. In our study, we expected that the incidence of grade 2 or worse neurotoxicity would be 20% in the GM1 group. On the basis of a one‐sided Fisher's exact test at a significance level of 2.5%, we needed a sample size of 98 patients per arm to provide 80% power to detect a 20% difference (20% in the GM1 group vs 40% in the placebo group) in the incidence of grade 2 or higher OIPN. The sample size in the study was inflated by 15% to account for patient withdrawal, ineligibility, poor compliance, or major protocol violations.

The differences in the percentage of patients experiencing grade 2 or greater chronic neurotoxicity according to NCI‐CTCAE 4.0 was compared between the two arms with the χ^2^ test. The EORTC QLQ‐CIPN20 sensory scale during chemotherapy was compared between the GM1 and placebo arms with the Wilcoxon rank sum test. The times to the onset of grade 2 or greater chronic neurotoxicity were compared between the two arms using Kaplan‐Meier survival curves and the log‐rank test. Acute neurotoxicity indicated by sensitivity to touching cold items, discomfort swallowing cold liquids, throat discomfort, and muscle cramps was summarized using descriptive statistics, and the *P* values were obtained by a repeated measures analysis of a variance model. Survival curves were generated according to the Kaplan‐Meier method, and disease‐free survival (DFS) was compared between the two groups with the log‐rank test.

## 3 RESULTS

### 3.1 Baseline characteristics

**Figure 1:**
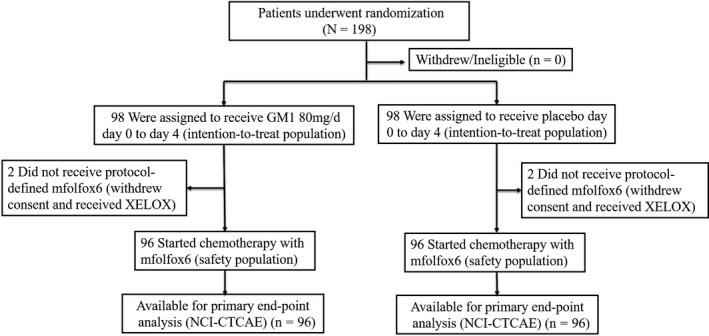
Flow diagram of this study

Between September 17, 2014, and September 5, 2017, 196 patients from 4 individual sites were enrolled and randomized into the GM1 and placebo arms. The baseline patient characteristics were equivalent in the two groups (Table S1). The flow chart of patient inclusion in the study is illustrated in the CONSORT diagram (Figure 1).

### 3.2 Chronic peripheral neurotoxicity

There was no statistically significant difference in the rate of NCI‐CTCAE grade 2 or worse neurotoxicity between the study arms. The incidence rates of NCI‐CTCAE grade 2 or worse neurotoxicity were 33.7% and 31.6% for the GM1 and placebo arms, respectively (primary end point; *P = *.76).

**Figure 2:**
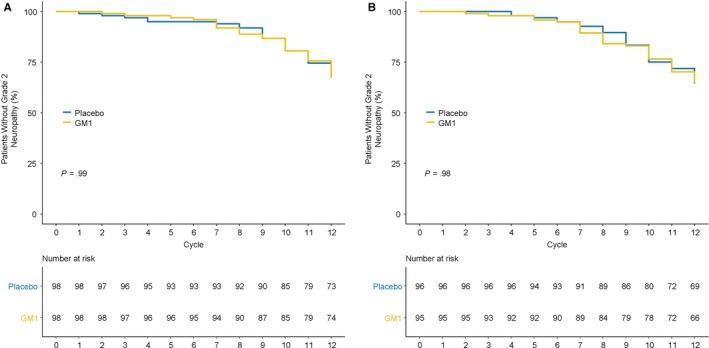
Time to grade 2 or worse oxaliplatin‐induced peripheral neuropathy, using (A) the National Cancer Institute Common Terminology Criteria for Adverse Events version 4 instrument (*P* = .99) and (B) the oxaliplatin‐specific neuropathy instrument (*P* = .98). GM1, monosialotetrahexosylganglioside

No substantial differences were observed for the time to grade 2 or worse neurotoxicity when OIPN was measured by the NCI‐CTCAE (Figure 2A, *P = *.99) or oxaliplatin‐specific neuropathy scale (Figure 2B, *P = *.98).

**Figure 3:**
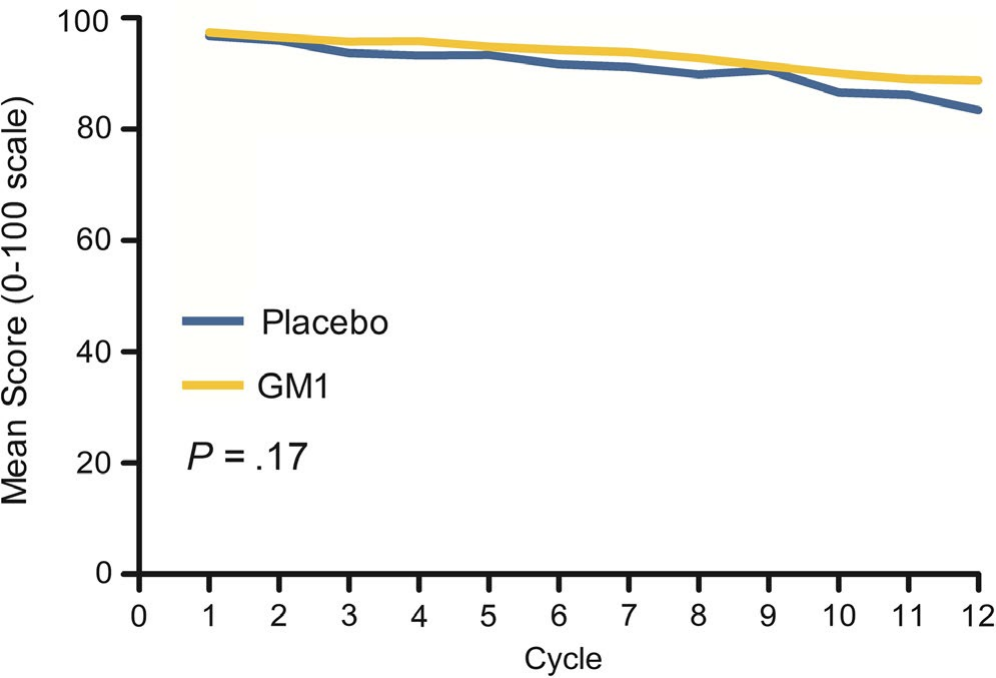
Oxaliplatin‐induced peripheral sensory neuropathy changes in the GM1 and placebo arms, as measured by the scale in the European Organization for Research and Treatment of Cancer Quality of Life Questionnaire‐Chemotherapy‐Induced Peripheral Neuropathy 20 instrument (Wilcoxon rank sum test, *P* = .17). GM1, monosialotetrahexosylganglioside

Similarly, there were no significant differences in neuropathy measured by the EORTC QLQ‐CIPN20 sensory neuropathy scale (Figure 3; *P = *.17 for comparing the GM1 arm with the placebo arm).

**Figure 4:**
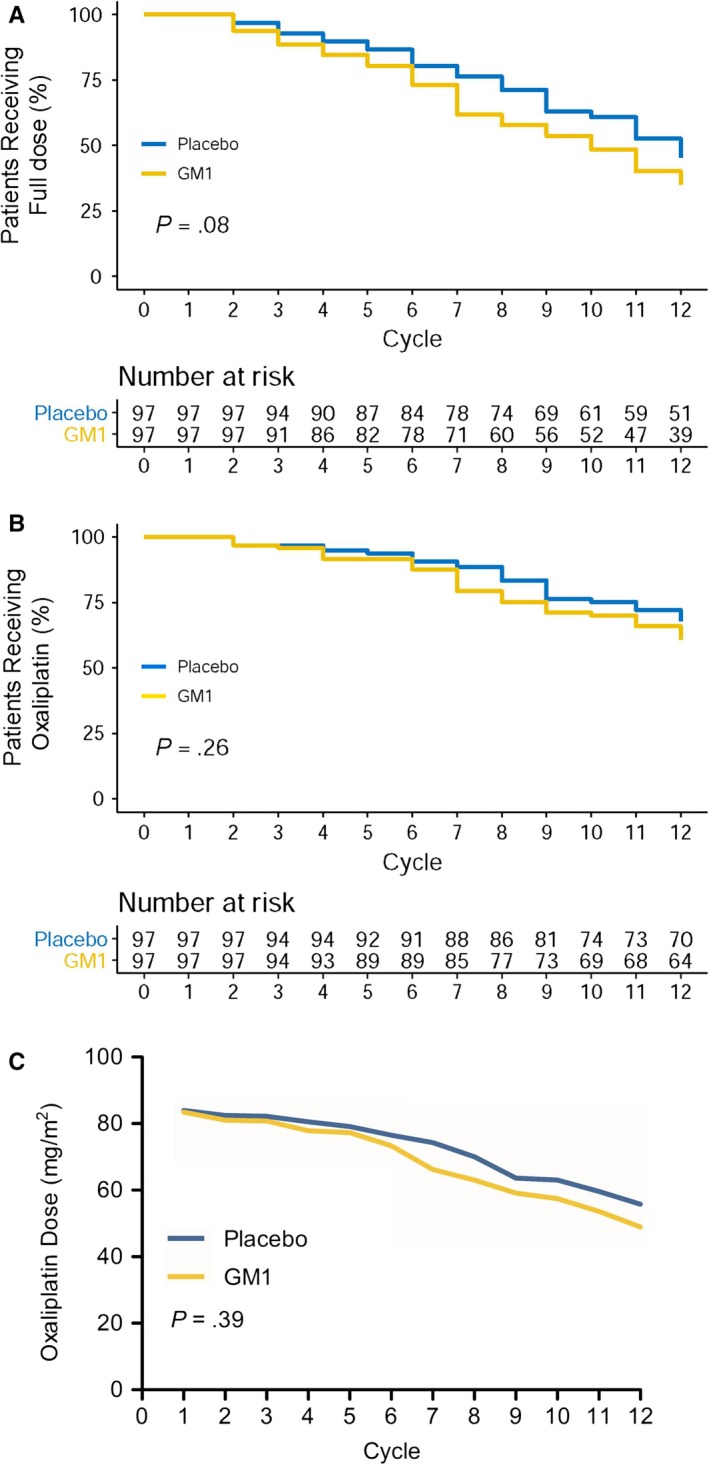
Percentages of patients in the GM1/placebo arm continuing to use (A) full doses of oxaliplatin over time (*P* = .08) or (B) oxaliplatin over time (*P* = .26). C, Mean doses of oxaliplatin in the GM1/placebo arms over time (*P* = .39). GM1, monosialotetrahexosylganglioside

For the dose intensity of oxaliplatin, there were no significant differences with regard to the rates of dose reduction or withdrawal of oxaliplatin between the two arms (GM1: 59.2% vs placebo: 46.9%, *P = *.08). The full doses of oxaliplatin over time, the use of oxaliplatin over time, and the mean oxaliplatin doses in the GM1 and placebo arms over time are shown in Figure 4A‐C. The median dose reduction cycles and median numbers of cycles to discontinuation for both arms are shown in Table S1.

### 3.3 Acute peripheral neurotoxicity

**Figure 5:**
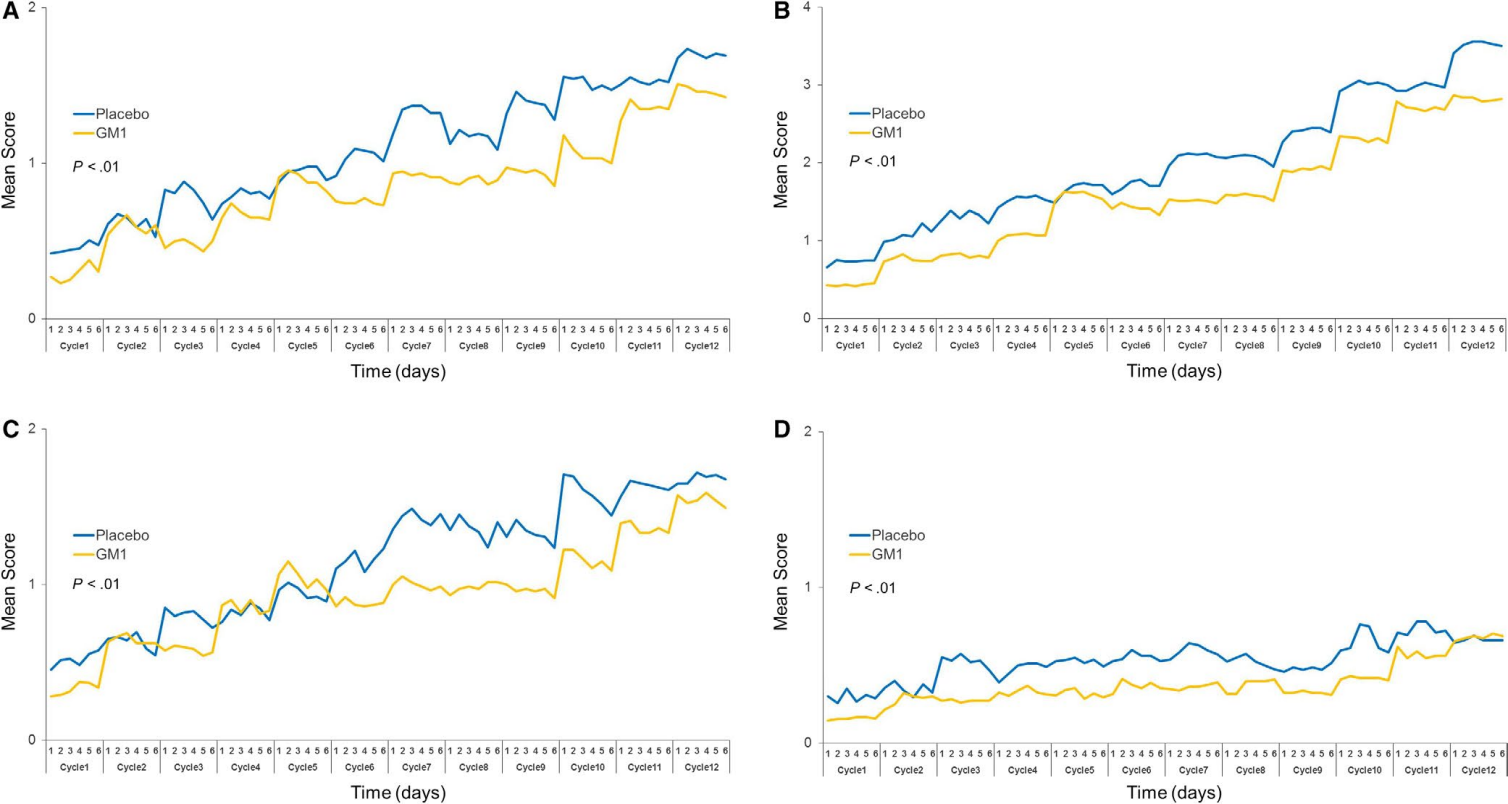
Acute neurotoxicity data regarding (A) discomfort swallowing cold liquids (*P* < .01), (B) sensitivity to touching cold items (*P* < .01), (C) throat discomfort (*P* < .01), and (D) muscle cramps (*P* < .01) in each study arm. GM1, monosialotetrahexosylganglioside

Patient‐reported acute neurotoxicity data regarding discomfort swallowing cold liquids, sensitivity to touching cold items, throat discomfort, and muscle cramps for 6 days after each cycle of oxaliplatin are shown in Figure 5. Interestingly, after a sequential analysis over 12 treatment cycles, it was clear that there were significant differences between the GM1 and placebo arms for each of the four symptoms. Figure 4 shows that GM1 substantially decreased the frequency of participant‐reported acute neurotoxicity symptoms with regard to the four symptoms mentioned above.

### 3.4 Evaluation of GM1 toxicity and survival

**Figure 6:**
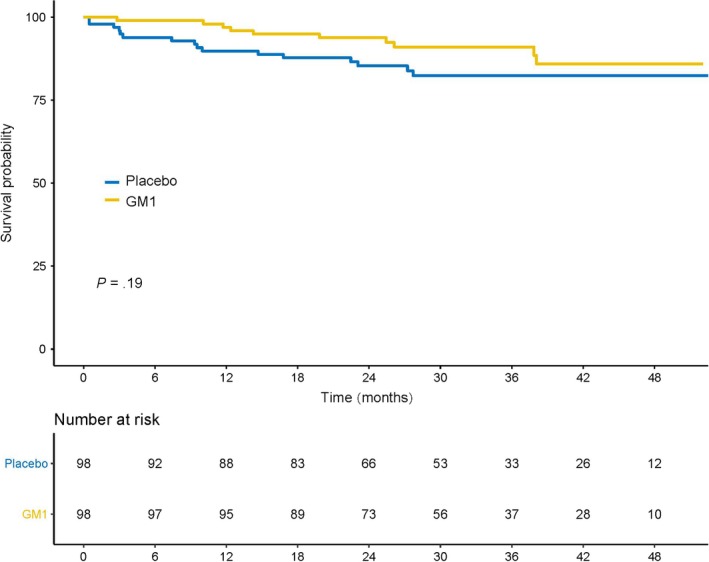
Kaplan‐Meier estimates of disease‐free survival in the GM1/placebo arms. GM1, monosialotetrahexosylganglioside

There were no differences in clinically apparent toxicity between the two study arms with regard to laboratory parameters, nausea, vomiting, mucositis, fatigue, hand‐foot syndrome, constipation, or diarrhea (Table S2). After a median follow‐up period of 31.6 months (IQR: 22.59‐42.87), the 3‐year DFS rates were 85% and 83% in the GM1 and placebo arms, respectively (HR 0.59; 95% CI, 0.27‐1.30; *P = *.19; Figure 6).

## 4 DISCUSSION

In the present phase III randomized, placebo‐controlled, double‐blind study, we did not find any significant differences between the GM1 and control arms with regard to the prevention of chronic neurotoxicity based on all the evaluation endpoints. However, interestingly, this trial showed that patients in the GM1 group were less troubled by acute neuropathy, with fewer patients in the GM1 group than in the placebo group reporting cold sensitivity, throat discomfort, and muscle cramps.

Oxaliplatin is an agent that is active against CRC and is used in both the adjuvant and palliative settings.^1^, ^2^ The most relevant side effect of oxaliplatin is peripheral neurotoxicity, which can manifest as either acute neuropathy or chronic neuropathy.^4^ Studies have shown that the mechanisms underlying acute and chronic neuropathy may be different.^13^

It has been reported that the mechanism underlying chronic neuropathy may be associated with damage to DRG neurons via energy failure or transport deficits.^9^, ^13^ NGF is an essential mediator of neuronal survival, differentiation, and maturation, including the activity of DRG neurons.^9^, ^10^, ^11^ A study found that oxaliplatin‐induced reversible sensory impairment was associated with a dose‐dependent reduction in the circulating level of NGF, indicating that NGF impairment plays a role in the neurotoxicity of oxaliplatin.^11^ GM1, a glycosphingolipid, is known to modulate neuronal plasticity^25^ and memory formation.^26^ Furthermore, GM1 has also been proposed as a neuroprotective agent against excitotoxic agents and ischemia^27^, ^28^ by enhancing the activity of NGF to promote the regeneration and recovery of nerves.^10^ Recently, two studies have suggested that GM1 can significantly reduce the incidence of OIPN^20^, ^21^; however, these retrospective studies with small sample sizes only evaluated the incidence of OIPN between the GM1 and control arms. No other indicators of neurotoxicity were measured. Another study conducted by Likun Zhou et al was designed to evaluate the efficacy of GM1 in gastrointestinal (GI) cancer patients with grade 2 or worse OIPN persisting during or after oxaliplatin‐based chemotherapy. The study revealed that 53% of patients in the GM1 arm and 14% of patients in the placebo arm achieved a ≥30% reduction in the modified EORTC QLQ‐CIPN20 (MCIPN20) score (RR = 3.85, *P* < .0001), suggesting that GM1 can effectively reduce OIPN in GI cancer patients.^19^ This phase II study aimed to determine the therapeutic effect of GM1, not the prevention of neurologic damage. In our present randomized, placebo‐controlled, double‐blind study, we assessed the efficacy of GM1 for preventing OIPN in CRC patients who received oxaliplatin‐based adjuvant chemotherapy. Furthermore, chronic cumulative neurotoxicity assessments were performed according to several separate methods. The acute neurotoxicity and the rates of dose reduction or withdrawal due to oxaliplatin‐induced neurotoxicity were also evaluated in both arms. Recently, a study conducted by Dr Yanhong Su et al^29^ have showed that the treatment with GM1 resulted in a reduction in the severity and incidence of taxane‐induced peripheral neurotoxicity after four cycles of taxane‐containing chemotherapy in patients with breast cancer. However, our results do not support the use of GM1 to prevent oxaliplatin‐induced cumulative neurotoxicity.

But interestingly, for the first time, the present study showed that patients in the GM1 group were less troubled than those in the placebo arm by symptoms of acute neuropathy, namely, cold sensitivity, throat discomfort, and muscle cramps, suggesting a protective effect of GM1 against acute symptoms. Acute neurotoxicity appears to be closely linked to functional alterations in voltage‐gated ion channels in nerve membranes and the neuromuscular junction.^30^ Some studies have suggested a prominent role for nuclear GM1 in the regulation of nuclear sodium‐calcium exchange^31^ and the prevention of seizures, Na^+^ and K^+^‐ATPase activity inhibition and oxidative stress induced by glutaric acid.^17^ Furthermore, it is reported that repeated episodes of acute hyperexcitability syndrome could eventually lead to the structural damage of neurons, eventually causing chronic neurotoxicity.^13^ Nevertheless, the results of our study did not show that the effect of GM1 on acute neuropathy can translate into a protective effect against chronic neuropathy. Clinical investigations previously attempted to use high doses of CaMg as a potential neuroprotectant against oxaliplatin‐induced acute and chronic neurotoxicity. However, although the N04C7 trials suggested a benefit of CaMg,^5^ the CONcePT trial and the phase III randomized study (N08CB/Alliance) both confirmed that intravenous CaMg does not appear to be an adequate solution to the problem of OIPN.^8^, ^32^

There were some limitations in our study. First, patients received GM1 80 mg per day from day 0 to day 4 during chemotherapy in our study mainly based on the previous retrospective study reports and convenience of clinical use.^19^ We did not perform a prior study on the optimal dosage and duration of GM1 to prevent OIPN, whether the dose and duration of GM1 therapy results in a different effect is still unknown. Second, this study did not shed light on the precise mechanisms that mediate the effect of GM1 on acute and chronic neurotoxicity.

In summary, the present study does not support the use of GM1 to prevent oxaliplatin‐induced cumulative neurotoxicity. However, compared to patients receiving the placebo, patients receiving GM1 were less troubled by the symptoms of acute neuropathy.

## CONFLICTS OF INTEREST

GM1 and the placebo were provided by the Qilu Pharmaceutical Co. Ltd, China. There are no other conflicts to disclose.

## DISCLOSURE OF PRIOR PRESENTATION

This study has been presented in part by Yu‐hong Li and De‐shen Wang, et al Abstract title: Phase III randomized, placebo‐controlled, double‐blind study of monosialotetrahexosylganglioside in prevention of oxaliplatin‐induced neurotoxicity in stage II/III colorectal cancer patients. Poster presentation at the 2019 Gastrointestinal Cancers Symposium in San Francisco, CA on January 17‐19, 2019. Abstract Perm ID: 674.

## AUTHOR CONTRIBUTIONS

All authors were involved in manuscript writing. Yu‐hong Li, Rui‐hua Xu and De‐shen Wang contributed to the conception and design of the study. De‐shen Wang, Zhi‐qiang Wang, and Gong Chen contributed to the collection and assembly of data. All authors contributed to the data analysis and interpretation. De‐shen Wang and Chuan‐bo Xie had access to the raw data and created the tables and figures. All authors had the opportunity to review the plan and outcome of analysis, participated in the preparation of this article, and provided final approval.
