# Supplementary material for: Phase III randomized, placebo‐controlled, double‐blind study of monosialotetrahexosylganglioside for the prevention of oxaliplatin‐induced peripheral neurotoxicity in stage II/III colorectal cancer
Source: Cancer Med. 2019 Nov 13;9(1):151–9. doi: 10.1002/cam4.2693 (PMC6943144; doi:10.1002/cam4.2693)
Supplement: Supplementary file 1 [file CAM4-9-151-s001.docx]

Table S1. Baseline patient characteristics and oxaliplatin dose modification by treatment arm.

|  | GM1 (N= 98) | | | | | Placebo (N= 98) | | | | |  |
| --- | --- | --- | --- | --- | --- | --- | --- | --- | --- | --- | --- |
| Characteristic | No. | % | Mean | SD | Median | No. | % | Mean | SD | Median | *P* |
| Age, years |  |  | 51.26 | 10.84 | 52.00 |  |  | 53.94 | 10.84 | 55.50 | 0.09 |
| Gender |  |  |  |  |  |  |  |  |  |  | 0.47 |
| Male | 53 | 54.1 |  |  |  | 58 | 59.2 |  |  |  |  |
| Female | 45 | 45.9 |  |  |  | 40 | 40.8 |  |  |  |  |
| Stage |  |  |  |  |  |  |  |  |  |  | 0.07 |
| II | 26 | 26.5 |  |  |  | 38 | 38.8 |  |  |  |  |
| III | 72 | 73.5 |  |  |  | 60 | 61.2 |  |  |  |  |
| Regimen |  |  |  |  |  |  |  |  |  |  | 1.00 |
| mFOLFOX6 | 96 | 98.0 |  |  |  | 96 | 98.0 |  |  |  |  |
| XELOX | 2 | 2.0 |  |  |  | 2 | 2.0 |  |  |  |  |
| Oxaliplatin reduction |  |  |  |  |  |  |  |  |  |  | 0.08 |
| Missing | 1 | 1.0 |  |  |  | 1 | 1.0 |  |  |  |  |
| Yes | 58 | 59.2 |  |  |  | 46 | 46.9 |  |  |  |  |
| No | 39 | 39.8 |  |  |  | 51 | 52.1 |  |  |  |  |
| Oxaliplatin reduction cycle |  |  | 8.82 | 3.437 | 10.0 |  |  | 9.70 | 3.12 | 12.0 | 0.065 |
| Oxaliplatin discontinued |  |  |  |  |  |  |  |  |  |  | 0.35 |
| Missing | 1 | 1.0 |  |  |  | 1 | 1.0 |  |  |  |  |
| Yes | 33 | 33.7 |  |  |  | 27 | 27.6 |  |  |  |  |
| No | 64 | 65.3 |  |  |  | 70 | 71.4 |  |  |  |  |
| Cycle discontinued |  |  | 10.26 | 2.877 | 12.0 |  |  | 10.69 | 2.539 | 12.0 | 0.268 |

Table S2. Selected Adverse Events, According to Treatment and Duration of Therapy.

|  | GM1, N (%) | | | Placebo, N (%) | | | *P* Value |
| --- | --- | --- | --- | --- | --- | --- | --- |
| Adverse Event | Grade 0 or 1 | Grade 2 | Grade 3 or 4 | Grade 0 or 1 | Grade 2 | Grade 3 or 4 |  |
| Haematopoietic system |  |  |  |  |  |  |  |
| Neutropenia | 46 (47.0) | 32 (32.7) | 20 (20.4) | 50 (51.1) | 31 (31.6) | 17 (17.3) | 0.968 |
| Thrombocytopenia | 81 (82.6) | 17 (17.3) | 0 (0) | 79 (80.7) | 17 (17.3) | 2 (2.0) | 0.308 |
| Anemia | 92 (93.9) | 6 (6.1) | 0 (0) | 92 (93.9) | 6 (6.1) | 0 (0) | 0.676 |
| Non-haematopoietic system |  |  |  |  |  |  |  |
| Diarrhea | 89 (90.8) | 7 (7.1) | 2 (2.0) | 85 (86.7) | 9 (9.2) | 5 (4.1) | 0.422 |
| Nausea | 79 (80.6) | 19 (19.4) | 0 (0) | 83 (84.7) | 15 (15.3) | 0 (0) | 0.469 |
| Vomiting | 91 (92.8) | 7 (7.1) | 0 (0) | 90 (91.8) | 7 (7.1) | 1 (1) | 0.656 |
| Mucositis | 90 (91.9) | 7 (7.1) | 1 (1) | 92 (93.9) | 6 (6.1) | 0 (0) | 0.690 |
| Fatigue | 89 (90.8) | 9 (9.2) | 0 (0) | 91 (92.9) | 7 (7.1) | 0 (0) | 0.464 |
| Hand–foot syndrome | 94 (96.0) | 4 (4.0) | 0 (0) | 92 (93.9) | 6 (6.1) | 0 (0) | 0.687 |
| Constipation | 96 (98.0) | 2 (2.0) | 0 (0) | 97 (99.0) | 1 (1.0) | 0 (0) | 0.745 |
